# 6-Methoxydihydroavicine is a benzophenanthridine alkaloid with anti-leukemia activity

**DOI:** 10.3389/fphar.2025.1621050

**Published:** 2025-06-24

**Authors:** Yingying Yang, Paul A. Spagnuolo

**Affiliations:** Department of Food Science, University of Guelph, Guelph, Canada

**Keywords:** benzophenanthridine alkaloid, 6-methoxydihydroavicine, acute myeloid leukemia, ROS, metabolism

## Abstract

**Background:**

Acute myeloid leukemia (AML) is an aggressive hematological malignancy with limited therapeutic options. Despite recent advances in targeted therapies, patients are still faced with poor survival outcomes. Thus, development of novel therapeutic agents with broad efficacy remains an urgent need.

**Methods:**

We conducted a natural compound library screen and identified 6-methoxydihydroavicine, a plant-derived benzophenanthridine alkaloid (BPA) derived from the genus of *Macleaya* - a perennial herb found in China, North America and Europe - as a potent compound that reduced AML cell viability. We evaluated its cytotoxicity in multiple AML cell lines and investigated its underlying mechanism of action using assays that probed mitochondrial function, and reactive oxygen species (ROS) production.

**Results:**

6-methoxydihydroavicine significantly reduced cell viability and induced caspase-mediated cell death in AML cell lines in a dose-dependent manner. Mechanistically, 6-methoxydihydroavicine triggered accumulation of mitochondrial ROS and disrupted electron transport chain (ETC) function.

**Conclusion:**

Our findings demonstrate that 6-methoxydihydroavicine exerts strong cytotoxic effects against AML cells through mitochondrial dysfunction and ROS-mediated apoptosis. As a natural, plant-derived compound with distinct anti-AML properties, 6-methoxydihydroavicine represents a promising candidate for further development as a therapeutic agent for AML.

## Introduction

Acute myeloid leukemia (AML) is a devastating hematological malignancy characterized by impaired hematopoiesis leading to uncontrolled proliferation and accumulation of undifferentiated myeloid precursors (myeloblasts) in the peripheral blood, bone marrow, and/or other tissues (Döhner et al., 2015; Papaemmanuil et al., 2016; Shimony et al., 2023). Recent data from the Surveillance, Epidemiology, and End Results Program shows that the 5-year survival rate for AML is 31.9% (National Cancer Institute, 2024), while in Canada, the adult 5-year net survival rate is as low as 21% (Statistics Canada, 2020). The standard “7 + 3” chemotherapy regimen, comprising 7 days of cytarabine and 3 days of anthracyclines, has remained largely unchanged since the 1980s (De Kouchkovsky et al., 2011; Newell and Cook, 2021). Although the development of targeted and immunotherapies has introduced new treatment options (Greiner et al., 2022), the prognosis for elderly patients remains poor with a 5-year survival rate of only 7% (Martínez-Cuadrón et al., 2021). AML patients often present with high-risk mutations and/or limited treatment tolerance leaving low-intensity chemotherapy as the main therapeutic strategy. Moreover, the molecular heterogeneity of AML further narrows therapeutic strategies, as existing targeted therapies benefit only a subset of patients harboring specific mutations (Greiner et al., 2022). Thus, new anti-AML drugs are needed to improve patient outcomes.

Natural products have played an important role in drug discovery and cytarabine, the front-line anti-AML therapeutic, was originally derived from natural sources (Chopra and Dhingra, 2021; Katz and Baltz, 2016; Hwang et al., 2019). Benzophenanthridine alkaloids (BPAs) are a class of isoquinoline compounds found widely in Papaveraceae, *Corydalis*, and Rutaceae families of plants. BPAs have demonstrated anti-tumour (Peng et al., 2023), anti-inflammatory (Lenfeld et al., 1981), anti-viral (Colombo and Bosisio, 1996), and anti-angiogenesis properties (Eun and Koh, 2004). Sanguinarine (SNG) and chelerythrine (CHE) are the most studied (Yang et al., 2012; Laines-Hidalgo et al., 2022), and a recent study showed SNG, CHE, and nitidine (NTD) have activity against gastric cancer cell lines (Liu et al., 2023).

An unbiased, systematic screen of an in-house natural product/nutraceutical library determined that 6-methoxydihydroavicine, a previously uncharacterized BPA, is a potent anti-AML compound that inhibits metabolic processes to impart selective cell death. 6-methoxydihydroavicine is commonly found in *Macleaya cordata* and *Zanthoxylum integrifoliolum*. While previous studies have reported anti-cancer activity in solid tumors such as pancreatic (Ma et al., 2022) and ovarian cancers (Zhang et al., 2023), its role in hematological malignancies remains largely unknown. In this study, we sought to investigate the anti-leukemia potential of 6-methoxydihydroavicine and elucidate its underlying mechanism in AML cells. Our results demonstrate that 6-methoxydihydroavicine exerts significant cytotoxicity in AML cell lines through induction of mitochondrial reactive oxygen species (ROS), inhibition of electron transport chain (ETC) activity, and activation of caspase-dependent cell death. These findings position 6-methoxydihydroavicine as a promising candidate for further development as a novel therapeutic agent against AML.

## Materials and methods

### Cell lines and materials

Ontario Cancer Institute AML2 (OCI-AML2; AML2), and OCI-AML3 (AML3) cell lines were cultured in Iscove’s Modified Dulbecco’s Medium (IMDM; Wisent) supplemented with 10% fetal bovine serum (FBS). The surrogate leukemia stem cell line, TEX, was grown in IMDM supplemented with 15% FBS (Sigma) and 20 ng/mL stem cell factor (SCF; Peprotech), 2 ng/mL interleukin-3 (Peprotech), and 2 mM L-glutamine (Thermo-Fisher). All media were supplemented with an antibiotic solution consisting of 200 μg of streptomycin and 200 units of penicillin per milliliter of media (Wisent). All cells were maintained in an incubator at 37°C with 5% CO_2_ and 95% relative humidity.

### Cytotoxicity analysis

To measure cell proliferation and viability, the 3-(4,5-dimethylthiazol-2-yl)-5-(3-carboxymethoxyphenyl)-2-(4-sulfophenyl)-2H-tetrazolium inner salt (MTS) reduction assay (Promega) and the 7-aminoactinomycin (7-AAD; Cayman Chemicals) dye were used. Cells (1.25 × 10^5^ cells/mL) were seeded in a 96-well plate and treated with serial dilutions of 6-methoxydihydroavicine at multiple time points. The untreated groups were incubated in media containing 0.06% dimethyl sulfoxide (matching the solvent volume used in the highest 6-methoxydihydroavicine concentration), serving as the negative control. Following treatment, for the MTS assay, cells were incubated with 20 μL of MTS for 2 h at 37°C and 5% CO_2_ and absorbance (490 nm) was measured using a SpectraMax M5 spectrophotometer (Molecular Devices; Sunnyvale, CA). For 7-AAD staining, cells were collected and resuspended in 250 μL of staining solution with 1 μg/mL 7-AAD, and the fluorescence was measured with the Guava EasyCyte 8HT Benchtop Flow Cytometer (Merck Millipore).

### Reactive oxygen species (ROS) quenching studies

To measure antioxidant effects on 6-methoxydihydroavicine cytotoxicity, leukemia cells (1.25 × 10^5^ cells/mL) were seeded in a 96-well plate and treated with increasing does of 6-methoxydihydroavicine in the absence or presence of 1 mM N-Acetylcysteine (NAC) for 72 h. Cell viability was then assessed using 7AAD (1 µg/mL) and fluorescence was measured using flow cytometry (Guava EasyCyte 8HT; EMD Millipore).

### Mitochondrial reactive oxygen species (ROS)

To assess mitochondrial ROS, leukemia cells were seeded in a 12-well plate (1.25 × 10^5^ cells/mL) and treated with increasing doses of 6-methoxydihydroavicine for 3 h. After treatment, cells were collected and stained with 5 μM MitoSOX Red probe (Invitrogen; M36008) according to the manufacturer’s protocol and fluorescence was measured by flow cytometry (Guava EasyCyte 8HT; EMD Millipore).

### Caspase-dependent cell survival assay

The pan-caspase inhibitor z-VAD-FMK (Z-VAD; R&D Systems, Minneapolis, MN) was used to study the role of caspase enzymes on 6-methoxydihydroavicine’s cytotoxicity. Here, leukemia cells were treated with 6-methoxydihydroavicine in the presence or absence of 50 μM Z-VAD. Following a 72 hour-incubation, cell viability was measured using the 7AAD assay and flow cytometry.

### Oxygen consumption rates

Oxygen consumption rates (OCRs) of leukemia cells were measured on the O2K Oxygraph (Oroboros) using substrate-uncoupler-inhibitor titration (SUIT) protocols (Gnaiger, 2020; Tcheng et al., 2021). Briefly, 5 × 10^6^ cells were treated with or without 1 μM 6-methoxydihydroavicine for 1 h. After treatment, cells were collected and permeabilized using 100 µg/mL digitonin (Sigma) and maintained in Mir05 buffer, as previously described (Tcheng et al., 2021; Makrecka-Kuka et al., 2015). Permeabilized cells were injected into the oxygraphy chamber and once equilibrated, 2.5 mM ADP, 5 mM pyruvate (PYR, Sigma), 10 mM glutamate (GLU; Sigma), and 0.5 mM malate (MAL, Sigma) were added to measure complex I (CI)-supported respiration. 250 nM rotenone (ROT, Sigma) was then added to inhibit CI respiration. To stimulate complex II (CII)-supported respiration, 10 mM succinate was injected. Finally, 250 nM antimycin A (AA; A8674; Sigma) was added to determine non-mitochondrial respiration. Oxygraph chambers were maintained at 37°C, and OCRs were calculated as the negative time derivative of oxygen concentration using the DatLab Software (Oroboros).

### Statistical analysis

Statistics were analyzed using GraphPad 8.0 Prism software. Results are presented as a mean ± standard deviation unless otherwise stated. Significance between values was determined by a paired, two-tailed t-test, two-way ANOVA, or by a one-way ANOVA paired with Dunnett’s *post hoc* analysis for between group comparisons. P < 0.05 was accepted as being statistically significant.

## Results

### 6-Methoxydihydroavicine is a plant-derived anti-AML agent

**FIGURE 1:**
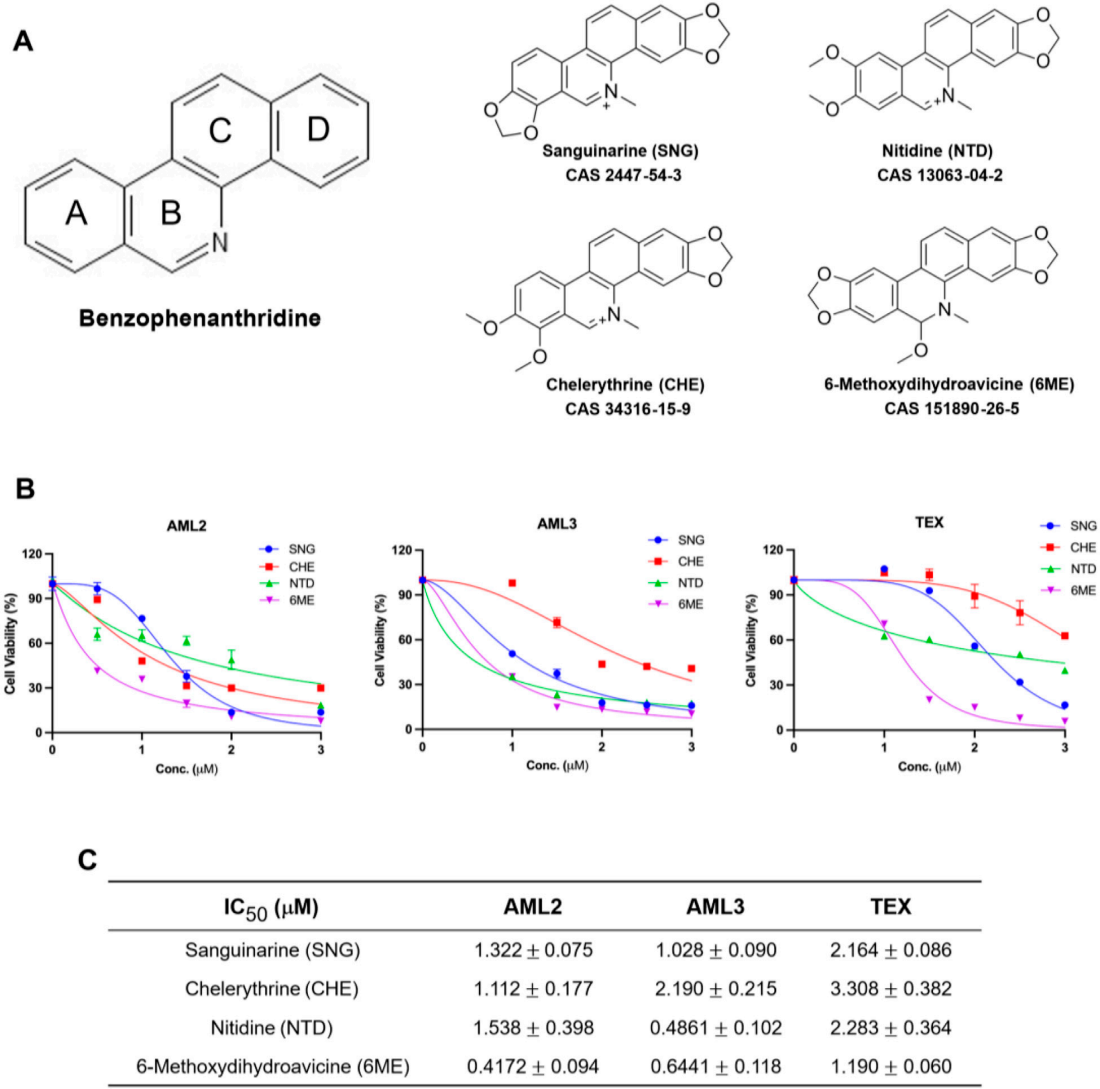
6-methoxydihydroavicine exhibits strong anti-AML potency. **(A)** The chemical structure of benzophenanthridine. **(B)** MTS assay, 7-AAD staining, and flow cytometry analysis of cell viability show the cytotoxic effects of SNG, CHE, NTD, and 6-methoxydihydroavicine in AML cell lines after 72-h treatment. **(C)** IC_50_ values of 6-methoxydihydroavicine are lowest among all tested BPAs, ranging from 0.42–1.19 µM. Data presented as % cell viability relative to untreated cells.

An in-house chemical library consisting of numerous plant-derived molecules screened against AML cells determined that 6-methoxydihydroavicine was a potent compound. Figure 1A shows the common molecular structure of BPA as well as 6-methoxydihydroavicine and the three additional BPAs included in this study: sanguinarine (SNG), chelerythrine (CHE), and nitidine (NTD). The activity of these BPAs was tested in AML2, AML3, and TEX leukemia cell lines using the MTS assay (Figure 1B), while the cytotoxicity of 6-methoxydihydroavicine was further confirmed using the 7-AAD assay and flow cytometry (Figure 1B). All four BPAs exhibited dose-dependent inhibitory effects on AML cells, with 6-methoxydihydroavicine showing the greatest potency with IC_50_ values ranging from 0.42 to 1.19 μM (Figure 1C). Cytarabine, a standard AML therapeutic, induces death in these cells at 5–10 μM (Tcheng et al., 2001).

### 6-Methoxydihydroavicine induced ROS participates in AML cell death

**FIGURE 2:**
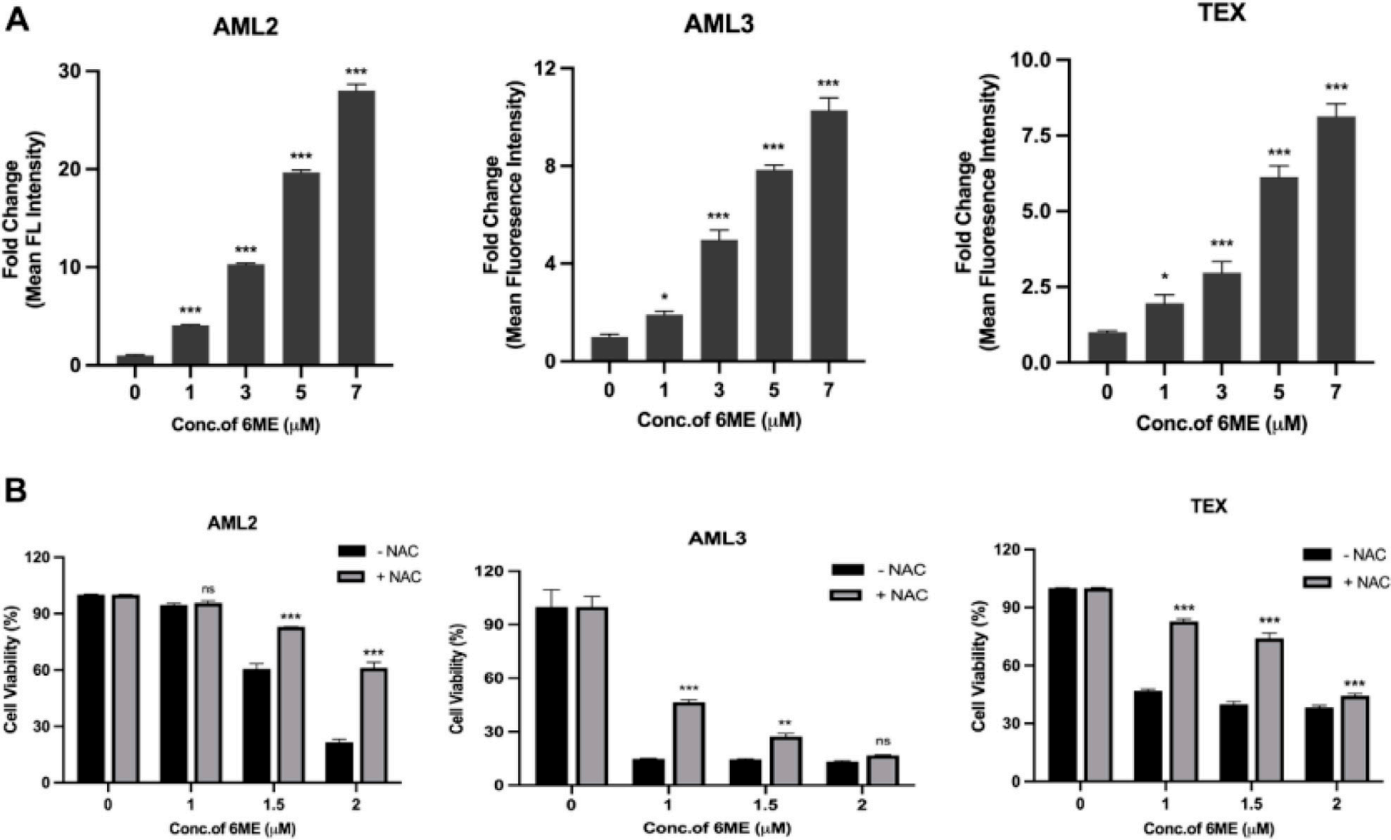
6-methoxydihydroavicine imparts anti-AML activity through ROS generation. **(A)** Mitochondrial ROS was measured following treatment with 6-methoxydihydroavicine for 3 h using MitoSOX red probe staining. **(B)** 6-methoxydihydroavicine activity was tested in the presence or absence of NAC (1 mM) in AML2, AML3, and TEX cells using 7AAD staining. Data presented as mean ± S.D., ns. not significant, *p < 0.05, **p < 0.01, ***p < 0.001 calculated using one-way ANOVA with Dunnett’s multiple comparisons test calculated in GraphPad Prism 8.0.

ROS, a class of highly reactive molecules that perform numerous biological activities including apoptosis induction (Nakamura and Takada, 2021), was measured in AML cells after 6-methoxydihydroavicine treatment. Using MitoSOX Red fluorogenic probes, 6-methoxydihydroavicine caused a dose-dependent accumulation of mitochondrial ROS (Figure 2A). To determine the functional significance of ROS in 6-methoxydihydroavicine-induced cytotoxicity, cells were also treated with NAC, which regenerates the potent antioxidant glutathione. NAC significantly abrogated 6-methoxydihydroavicine-induced cytotoxicity (Figure 2B), suggesting a functional role of ROS.

### 6-Methoxydihydroavicine imparts anti-AML activity via caspase-mediated death and mitochondrial dysfunction

**FIGURE 3:**
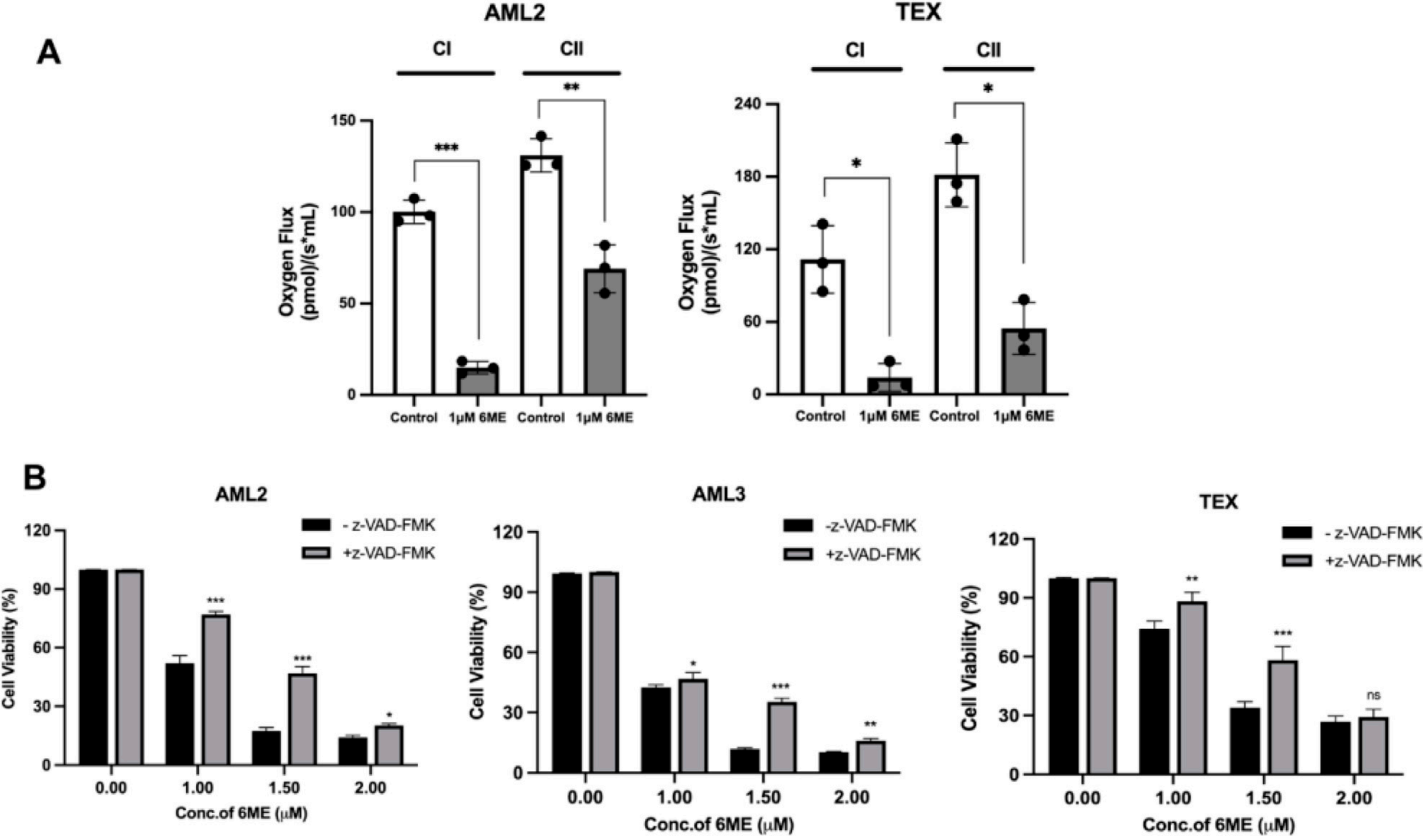
6-methoxydihydroavicine-induced AML cell death via modulation of caspase activity and mitochondrial dysfunctions. **(A)** Oxygen consumption rates (OCRs) of complexes I and II were measured AML2 and TEX cells treated with 1 μM 6-methoxydihydroavicine for 1 h following a well-established substrate-uncoupler-inhibitor titration (SUIT) protocol using the Oroboros 2K oxygraphy. **(B)** 6-methoxydihydroavicine activity was tested in the presence or absence Z-VAD (50 μM) in AML2, AML3, and TEX cells using 7AAD staining. Data presented as mean ± S.D., ns. not significant, *p < 0.05, **p < 0.01, ***p < 0.001 calculated using an unpaired t-test or one-way ANOVA and a Dunnett’s multiple comparisons test in GraphPad Prism 8.0.

Mitochondria are a primary source of cellular ROS (Turrens, 2003). Electrons leaking from the mitochondrial electron transport chain (ETC) can react with oxygen and produce ROS. To investigate effects on, ETC, function, respiration rates of complexes I and II were measured in 6-methoxydihydroavicine-treated AML2 and TEX cells using a well-established SUIT protocol and respirometry. 6-methoxydihydroavicine significantly inhibited respiration of both complexes compared to untreated groups in both AML2 and TEX cells (Figure 3A), indicating that 6-methoxydihydroavicine impacts mitochondrial bioenergetics.

Finally, to assess whether caspase enzymes are involved in cell death, we co-incubated 6-methoxydihydroavicine with the pan-caspase inhibitor Z-VAD (50 μM). After incubation for 72 h, Z-VAD significant reduced 6-methoxydihydroavicine-induced reductions of AML cell viability (Figure 3B), implicating the role of caspase activity in 6-methoxydihydroavicine-induced cell death.

## Discussion

A high-throughput screen identified 6-methoxydihydroavicine as a novel anti-AML compound, and validation studies determined that this alkaloid, derived from a plant found in North America, was capable of inducing cytotoxicity in AML through metabolic alterations. In this study, 6-methoxydihydroavicine triggered AML cell death through ROS induction, caspase activity, and mitochondrial dysfunction.

6-methoxydihydroavicine is a BPA with reported anti-cancer activity in solid tumors (Ma et al., 2022; Zhang et al., 2023); however, its role in AML and its mechanism of action in hematologic malignancies remain largely unknown. In pancreatic and ovarian cancer cells, 6-methoxydihydroavicine activates the RIPK1/caspase axis leading to disruption of oxaloacetic acid metabolism (Ma et al., 2022) and alters mitochondrial respiration to induce MAPK-mediated apoptosis (Ma et al., 2022; Zhang et al., 2016). Previous work by our group showed that 6-ME bound to and decreased activity of PPAR-γ leading to decreased fatty acid oxidation (FAO) and selective death of AML cells. In the current study, we extended these findings and build upon the work of others to show that 6-methoxydihydroavicine-induced cell death is associated with ROS and caspase activity. The observed cytotoxicity of 6-methoxydihydroavicine is related to elevated mitochondrial ROS, which was mitigated when co-incubated with the glutathione regenerating molecule NAC (Figure 2B). These results are in line with previous findings that FAO inhibition, through decreased NADPH leading to reduced glutathione production, results in ROS-induced cell death that was mitigated by NAC co-incubation (Lee et al., 2015; Pike et al., 2011). In this study, 6-methoxydihydroavicine also decreased CI and CII activity and CI or II inhibition results in ROS-mediated death of cancer cells (Basit et al., 2017; Li et al., 2003; Quinlan et al., 2012; Dröse, 2013; Guzy et al., 2008; Roma et al., 2022), which aligns with a previous report that targeting oxidative stress could be an effective approach for cancer treatment (Ma et al., 2018). Whether this change in complex activity is related to PPARγ targeting or an off-target effect will be the focus of future studies. Additional work is also needed to confirm the functional importance of CI and CII in 6-methoxydihydroavicine-induced cytotoxicity as well as the link between complex inhibition, PPAR inhibition and ROS generation.

Plants are a significant source of clinically relevant anti-cancer drugs. Indeed, the most common clinical AML therapeutic, cytarabine, was derived from *Cryptotheca crypta,* a Caribbean sponge (Schwartsmann et al., 2001). BPAs, which are isolated form Papaveraceae, *Corydalis*, and Rutaceae families of plants, are noted for their anti-inflammatory, anti-tumor, and anti-bacterial activities (Peng et al., 2023). From the genus of *Macleaya* (Papaveraceae), these plants are primarily found in China, Europe and North America and have been used historically for medicinal effects. SNG exerts an anti-proliferative activity on murine melanoma cells and A375 human melanoma xenografts *in vitro* and *in vivo* (De Stefano et al., 2009). Mechanically, SNG induces H_2_O_2_-dependent cell ferroptosis in human cervical cancer (HeLa) cells by downregulating SLC7A11 and glutathione (Alakkal et al., 2022). Other BPAs, like CHE, inhibit non-small cell lung cells by downregulating β-Catenin (Heng and Cheah, 2020), while NTD suppresses epithelial mesenchymal transformation and glioma stem cells by targeting the JAK2/STAT3 signaling pathway (Jia et al., 2021). Moreover, a synthetic BPA, NK109, demonstrated strong anti-leukemia activity (Nakanishi et al., 1999), with its metabolites showing reduced toxicity to host cells while retaining significant anti-tumor effects in clinical trials (Nakanishi et al., 2000). Notably, several natural BPAs show superior activity compared to marketed drugs *in vitro*. Burgenine is more potent than doxorubicin in drug-resistant CEM/ADR5000 cell lines (Sandjo et al., 2014), while oxynitidine derivatives display greater inhibitory effects against DU145 prostate cancer cells compared to camptothecin (Tang et al., 2019). While clinical data on natural BPAs remain limited, early-stage trials and preclinical evidence suggest a favorable therapeutic window (Peng et al., 2023; Tavares de et al., 2014; Xu et al., 2025; Pica et al., 2012), with certain compounds demonstrating selective cytotoxicity against cancer cells and manageable toxicity profiles in animal models. In this study, other BPAs demonstrated potency with low IC50 values and 6 ME was selected as the lead compound, as it was consistently the most potent. Future studies will determine which structural features contribute to 6 ME’s superior activity. These findings collectively highlight the therapeutic promise of BPAs and support their further investigation as potential anti-cancer agents.

In summary, found in plants growing in North America, 6-methoxydihydroavicine is a novel compound belonging to the BPA family that exhibited potent anti-AML activity. Mechanistically, it induces mitochondrial ROS, impairs CI and CII respiration, and imparts caspase-dependent cell death. These findings provide new insights into 6-methoxydihydroavicine as a potential anti-leukemia therapeutic.

## Data Availability

The raw data supporting the conclusions of this article will be made available by the authors, without undue reservation.
